# A Colorimetric Dermal Tattoo Biosensor Fabricated by Microneedle Patch for Multiplexed Detection of Health‐Related Biomarkers

**DOI:** 10.1002/advs.202103030

**Published:** 2021-11-01

**Authors:** Rongyan He, Hao Liu, Tianshu Fang, Yan Niu, Huiqing Zhang, Fei Han, Bin Gao, Fei Li, Feng Xu

**Affiliations:** ^1^ The Key Laboratory of Biomedical Information Engineering of Ministry of Education Xi'an Jiaotong University School of Life Science and Technology Xi'an 710049 China; ^2^ Bioinspired Engineering and Biomechanics Center (BEBC) Xi'an Jiaotong University Xi'an 710049 China; ^3^ Key Laboratory of Thermo‐Fluid Science and Engineering of Ministry of Education School of Energy & Power Engineering Xi'an Jiaotong University Xi'an 710049 China; ^4^ Department of Endocrinology Tangdu Hospital Air Force Military Medical University Xi'an 710038 China

**Keywords:** health monitoring, microneedle patch, point‐of‐care testing, skin interstitial fluid, wearable biosensors

## Abstract

Detection of biomarkers associated with body conditions provides in‐depth healthcare information and benefits to disease management, where the key challenge is to develop a minimally invasive platform with the ability to directly detect multiple biomarkers in body fluid. Dermal tattoo biosensor holds the potential to simultaneously detect multiple health‐related biomarkers in skin interstitial fluid because of the features of minimal invasion, easy operation, and equipment‐free result reading. Herein, a colorimetric dermal tattoo biosensor fabricated by a four‐area segmented microneedle patch is developed for multiplexed detection of health‐related biomarkers. The biosensor exhibits color changes in response to the change of biomarker concentration (i.e., pH, glucose, uric acid, and temperature), which can be directly read by naked eyes or captured by a camera for semi‐quantitative measurement. It is demonstrated that the colorimetric dermal tattoo biosensor can simultaneously detect multiple biomarkers in vitro, ex vivo, and in vivo, and monitor the changes of the biomarker concentration for at least 4 days, showing its great potential for long‐term health monitoring.

## Graphical abstract

Simultaneous detection of multiple biomarkers provides in‐depth healthcare information, where the key challenge is to develop a minimally invasive platform. To this end, a colorimetric dermal tattoo biosensor fabricated by a four‐area segmented microneedle patch is developed. The biosensor exhibits color changes in response to biomarker concentration variations, showing its great potential for health monitoring.

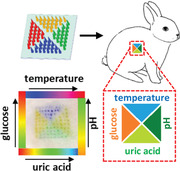

## 1 Introduction

Monitoring of health‐related biomarkers, such as biochemical molecules (e.g., glucose and uric acid) and biophysical signals (e.g., temperature), can provide in‐depth personal information in prediction, screening, diagnosis of diseases and healthcare management. Currently, various platforms have been developed to monitor health‐related biomarkers.^[^ ^1^ ^]^ For instance, wearable wristbands integrated with flexible substrates and electrochemical biosensors could monitor metabolites in sweat for hours.^[^ ^2^ ^]^ To enhance the wearing comfort, epidermal tattoo biosensors made of micro‐ or nano‐scale films are closely attached to the skin to detect biophysical signals or biochemical molecules.^[^ ^3^ ^]^ In these strategies, biochemical molecules are detected in biofluids exuded or secreted onto human body surface (e.g., sweat, saliva, or tear). However, the difference in compositions between these secreted biofluids and blood, time lag in detection, and environmental contamination limit the applications of these sensors in health and disease management.^[^ ^4^ ^]^ Implantable sensors enable to directly detect molecules from biofluid in body (e.g., blood, interstitial fluid (ISF) and cerebrospinal fluid). But integrated circuit and wireless signal generator make the implantable sensors large in dimensions (cm level),^[^ ^5^ ^]^ and potential issue of injury when implanting and removing the sensors are involved. Therefore, there is still an unmet need for developing minimally invasive platforms with the ability to directly detect health‐related biomarkers in body fluid.

Dermal tattoo is a body art modification by injecting pigments into skin dermal layer.^[^ ^6^ ^]^ The replacement of traditional tattoo inks with colorimetric sensing reagents can contribute to a quantitative sensing platform to detect health‐related biomarkers in skin ISF. Since skin ISF transports metabolites between capillaries and cells, supporting tissue with nutrients and eliminating waste products,^[^ ^7^ ^]^ it provides highly similar metabolic information with that in blood and less time lag from blood compared with other biofluids, making it a promising sample source to monitor health‐related biomarkers.^[^ ^8^ ^]^ In addition, dermal tattoo biosensors are formed under the skin stratum corneum, which provides a strong natural physical barrier to protect colorimetric reagents inside skin from external environment, and thus enable stable detection without compromises to contamination of skin surface (e.g., sweat and rainwater). Such a physical barrier can also prolong the device service life and further facilitate the applications of dermal tattoo biosensors in long‐term monitoring. Therefore, a dermal tattoo biosensor that could detect biomarkers in skin ISF can be used to reflect individual health conditions.

Dermal tattoo biosensors can be fabricated using commercial tattoo guns and successfully detect metabolites and electrolytes (e.g., pH, glucose, albumin, Na^+^, and K^+^) ex vivo.^[^ ^9^ ^]^ However, it is difficult to achieve controllable and repeatable injection of tattoo ink with consistent depth and angle using a hand‐held commercial tattoo gun. Uniform colorimetric reading is closely associated with the depth of colorimetric sensing reagents embedded in skin,^[^ ^10^ ^]^ and inconsistent injection depth may induce deteriorated accuracy and reliability of sensing results. The angle at which it penetrates the skin affects the ink distribution in the dermis,^[^ ^11^ ^]^ where vertical injection makes the ink distribution more concentrated and the color clearer compared with oblique injection. Besides, because a tattoo gun can only be loaded with detection reagents for one biomarker at a time, commercial guns‐made dermal tattoo biosensors need complex and multiple‐step operation to detect several biomarkers. In recent years, microneedle patches have been widely investigated for biomedical applications including diagnosis and drug delivery.^[^ ^12^ ^]^ Microneedle patches are appropriate tools to pattern dermal tattoo biosensor owing to their simple operation, and consistency in penetration depth and angle.^[^ ^13^ ^]^ In addition, tattoo patterns can be easily designed and flexibly tuned using microneedle patches, and they can realize simultaneous detection of multiple biomarkers in one patch by loading different reagents inside the needle carrier of different regions on the patch. Moreover, microneedle patches reduce exposure of sensing reagents to air and avoid contamination of sensing reagents on the skin surface when delivering reagents into dermis. This can be explained by that the sensing reagents are loaded in microneedle tips, which directly penetrate the skin when used, and thus the reagents do not stay on the skin surface, avoiding their oxidation by air or contamination by skin. In addition, microneedles can provide as many as 100 sensing results in a single test and averaging the many obtained data can further mitigate errors from manual operation, which is especially intractable for the hand‐held tattoo gun. Compared with the tattoo gun used to fabricate dermal sensors in previous studies,^[^ ^9^ ^]^ microneedle patches are minimally invasive, painless, more user‐friendly, and reliable.^[^ ^8a^ ^]^ Hence, dermal tattoo biosensor fabricated by microneedle patches has the potential to be an ideal platform to achieve detection of multiple health‐related biomarkers.

In this work, we developed a colorimetric dermal tattoo biosensor fabricated by a microneedle patch for simultaneously detecting multiple health‐related biomarkers ex vivo and in vivo. To demonstrate the capability of the biosensor for multiplexed detection, four typical biomarkers (i.e., pH, glucose, uric acid, and body temperature) related to chronic diseases were chosen. The colorimetric reaction reagents were injected into dermis by microneedle patches with minimal invasion to form dermal tattoo biosensors. The biosensor exhibits color changes in response to variations in pH, glucose, uric acid, and body temperature, which can be directly read by naked eyes for qualitative detection or captured by a smartphone camera for further semi‐quantitative analysis. We also demonstrated the successful realization of the biosensor for simultaneously monitoring the pH value and temperature of rabbit's skin for 4 days in vivo, indicating the great potential of the developed tattoo biosensor for long‐term health monitoring.

## 2 Results and Discussion

### 2.1 Fabrication of Colorimetric Dermal Tattoo Biosensor Based on Patterned Microneedle Patches

**Figure 1:**
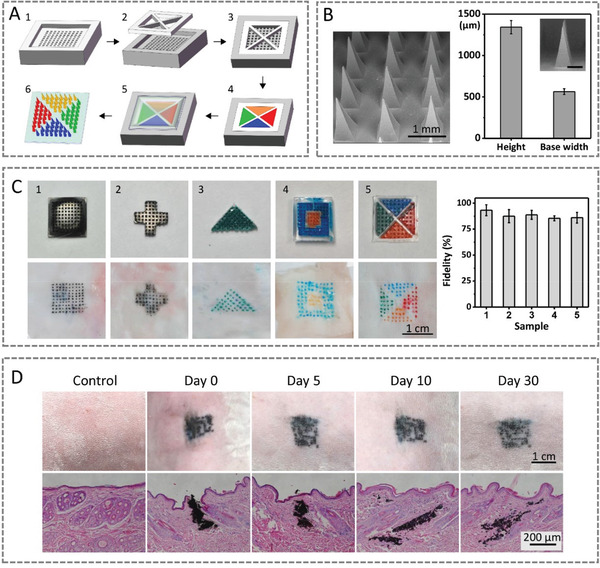
Characterizations of the patterned microneedle for fabricating colorimetric dermal tattoo biosensors. A) Schematic illustration of fabricating patterned microneedle patches. B) Surface morphology and dimension of tattoo microneedle patches. C) Patterned dermal tattoos fabricated by microneedle patches and fidelity of tattoos in skin compared with designed tattoo patterns. D) Images (upper row) and H&E slice staining (lower row) of skin before and after inserting microneedle dermal tattoo biosensors in rabbit's skin for 0–30 days.

A desirable patterned microneedle patch as dermal tattoo biosensors should have morphologically intact needle tips to penetrate into the skin dermal layer, release colorimetric reagents by dissolving the microneedles tips, and form designed patterns with high fidelity in skin dermis.^[^ ^8^, ^9^, ^14^ ^]^ To meet these demands, we fabricated HA‐based microneedle patches with designed patterns (**Figure** **1**A). The scanning electron microscope (SEM) images show that the microneedles have an intact pyramid structure with a height of 1343.6 ± 80.1 µm and a base length (i.e., the side length of the bottom square) of 565.3 ± 35.2 µm (Figure 1B). The skin is composed of epidermis and dermis with thickness of ≈76.9–267.4 µm and ≈2115–5888 µm, respectively.^[^ ^15^ ^]^ It indicates that the fabricated microneedles are long enough to penetrate through the epidermis and reach the dermal layer for releasing sensing reagents which can react with the biomarkers in ISF. This strategy is helpful to avoid injuring blood vessels and nerves locating at the deeper dermis, without causing bleeding and pain.^[^ ^16^ ^]^

To fabricate the colorimetric dermal tattoo biosensors, we inserted patterned microneedle patches into rabbit skin, where multiple dyes were used to mimic the colorimetric sensing reagents (Figure 1C). We observed that the dyes are released from the patterned microneedle patches into dermis, forming dermal tattoo. After removing the microneedle patches from skin, all the microneedle tips become shorter (to one‐third of their original heights) and blunt (Figure S1, Supporting Information), indicating the dissolution of the tips into the skin. We further calculated the fidelity of patterns by measuring the ratio of the overlaps area (i.e., the skin dermal tattoo overlaps the designed pattern) to the area of the designed pattern. The fidelity of all dermal tattoos is over 85%, and decreases as the pattern complexity increases. This was further confirmed by the clear tattoo patterns without cross‐contamination of dyes with different colors (Figure 1C). The results demonstrate that the detection area of different biomarkers can be separated, providing potential for simultaneous detections of multiple biomarkers. To study the location of the formed tattoo in the skin, we sliced the skin tissue with the tattoo and stained the skin with hematoxylin‐eosin (H&E) dyes. We observed that there are clear pinholes and tattoo ink in the skin dermis (for at least 30 days), indicating the successful penetration of the microneedles to skin dermis and the tattoo ink stays in dermal layer without infiltration into epidermis for at least 30 days (Figure 1D).

### 2.2 Feasibility of Colorimetric Dermal Tattoo Biosensors for Detecting Four Biomarkers In Vitro

**Figure 2:**
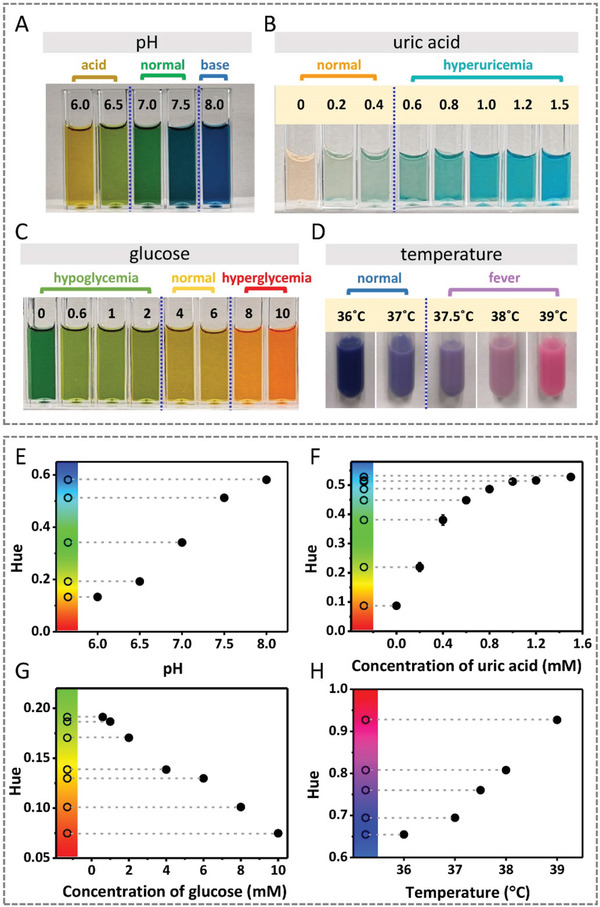
Detections of the four biomarkers (i.e., pH, uric acid, glucose and temperature) in vitro using colorimetric sensing reagents of dermal tattoo biosensors. A–D) Images of colorimetric detection of pH (A), uric acid (B), glucose (C), and temperature (D) in vitro. E–H) The relationship between the Hue values extracted from the images in (A–D) and the levels of pH (E), uric acid (F), glucose (G) and temperature (H). Color bar of Hue is presented on the left of figures.

The ability of the colorimetric dermal tattoo biosensors for healthcare monitoring was demonstrated by detecting four typical chronical disease‐related biomarkers, including three health‐related biochemical biomarkers (i.e., pH, uric acid, and glucose) and one biophysical signal (i.e., temperature) (**Figure** **2**). These four biomarkers are typical detection indicators in chronical disease monitoring.^[^ ^17^ ^]^ First, three pH indicator reagents (i.e., thymol blue, methyl red, and phenolphthalein) were used as the pH biosensor to detect pH values in vitro. We observed a distinct color change in phosphate buffer solution (PBS) solution with pH range of ≈6.0–8.0 (the fluctuation range of human skin ISF^[^ ^18^ ^]^) (Figure 2A), in which the solution shows yellow at pH 6.0, turns to green and then blue with increasing pH. We can distinguish pH difference as low as 0.5 by naked eyes in this range, which can achieve semi‐quantitative detection of pH value in vitro. To further prove the ability of the pH sensor for long‐term use, we assessed the stability of pH sensors by measuring their absorbance of solutions for 6 days. We observed that the peak values of the absorbance are stable and maintain over 82% of the original value after 6 days (Figure S2, Supporting Information), indicating the reliably of the pH sensors for at least 6 days without significant fading.

Second, considering that high uric acid level (hyperuricemia, >0.45 mm) increases the risk of disease,^[^ ^19^ ^]^ we used the enzymatic reaction‐based colorimetric method to detect the uric acid concentration based on the reaction that uric acid oxidizes to produce allantoin and hydrogen peroxide under catalysis of uricase (Figure 2B). We observed that the solution color changes from light orange (the color of horseradish peroxidase) to blue with increasing uric acid concentration in the range of ≈0–1.5 mm, which is due to that the produced hydrogen peroxide oxidizes 3,3′,5,5′‐tetramethylbenzidine (TMB) to form a blue‐colored oxidation product under peroxidase catalysis.^[^ ^20^ ^]^ Thus, we can easily distinguish the normal uric acid levels (orange and light green) and hyperuricemia (green and blue) with naked eyes for semi‐quantitative detection of uric acid levels.

**Figure 3:**
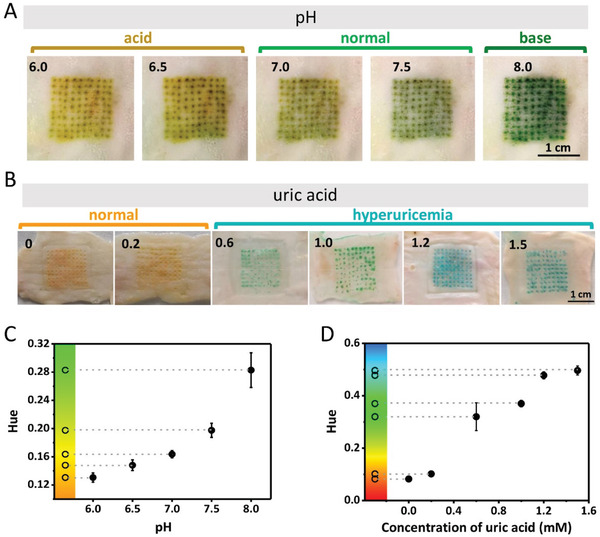
Detections of pH and uric acid ex vivo using colorimetric dermal tattoo biosensors. A) Colorimetric detection of pH ex vivo in the range of 6.0 to 8.0. B) Colorimetric detection of uric acid ex vivo in the range of 0 to 1.5 mm. C,D) The Hue values extracted from the images in (A) and (B) as a function of pH (C) and uric acid concentration (D).

Third, we detected glucose by converting glucose level to gluconic acid under the catalysis of glucose oxidase and the produced gluconic acid changes solution pH, which can be detected by the pH biosensor (**Figure** **3**C). We observed that the solution shows green at hypoglycemia (<4 mm), turns to yellow at normal glucose level (≈4–6 mm), and red at hyperglycemia (>8 mm), and the glucose sensing reaction completes within 10 min, and the color remains stable for 60 min (Figure S3, Supporting Information), indicating that the low, normal, and high glucose level can be distinguished with the naked eyes.

Fourth, temperature‐responsive solution was used to detect temperature in vitro. We observed that the solution changes to blue at 36 °C, and turns to purple and magenta at ≈37–38 °C and 39 °C, respectively (Figure 2D). These results indicate that the tattoo temperature biosensor shows dark blue, purple, and magenta at normal temperature (≈36–37 °C), slight fever (≈37.5–38 °C) and severe fever (39 °C), respectively, which can be distinguished by naked eyes. The high critical temperature of the developed sensor is set to be 39 °C because the temperature tattoo sensor in our work measures the temperature of skin surface, which is about 1 °C lower than rectal temperature.^[^ ^21^ ^]^ When the skin temperature detected by our sensor exceeds 39 °C, the corresponding rectal temperature can reach 40 °C, which is dangerous and can cause serious, irreversible long‐lasting brain damage.^[^ ^22^ ^]^ Patients with that high fever are suggested to go to the hospital for further accurate diagnosis and treatments.

To further prove the accuracy of distinguishing colors with naked eyes in reading results and semi‐quantify the concentration and degree of biomarkers, we extracted the Hue values of the HSV color space (Hue, Saturation, and Value) from the images. Compared with the shade of color, which is commonly used in colorimetric detection studies and analyzed by gray value, Hue is relatively stable since it is determined by the pigments produced by colorimetric reaction (Figure S4, Supporting Information). The results in Figure 2 show that the Hue values are correlated to the levels of biomarkers in a certain range. For pH, uric acid, and temperature sensors, Hue values increase with the increase of corresponding biomarkers. While for glucose sensor, Hue values decrease with the increase of concentration of glucose (Figure 2E–H). The color bars of the Hue in the left of each figure were used to confirm the color changes as observed by the naked eyes, which is conducive to check the correctness of hue value extraction and analysis.

### 2.3 Detection of Four Biomarkers Ex Vivo Using Colorimetric Dermal Tattoo Biosensors

To further confirm the detecting capability, we utilized the colorimetric dermal tattoo biosensors on a piece of isolated rabbit skin and detected four biomarkers ex vivo (Figure 3). Specifically, we inserted the microneedle patches loaded with pH sensing reagents into the rabbit skin to form tattoo pH sensor and soaked the skin in PBS solution with different pHs (≈6.0–8.0). We observed that tattoo pH sensor in skin shows yellow at pH 6.0 and then turns to green with increasing pH (Figure 3A).

To detect uric acid ex vivo, we fabricated dermal tattoo uric acid sensor in rabbit skin and then soaked it in solution with varying uric acid concentrations (≈0–1.5 mm). We observed that the tattoo turns from orange to green and blue along with the increase of the uric acid concentration (Figure 3B). The analysis of the tattoo color further confirmed that the monolithic Hue increases with increasing pH and uric acid concentrations (Figure 3C,D).

**Figure 4:**
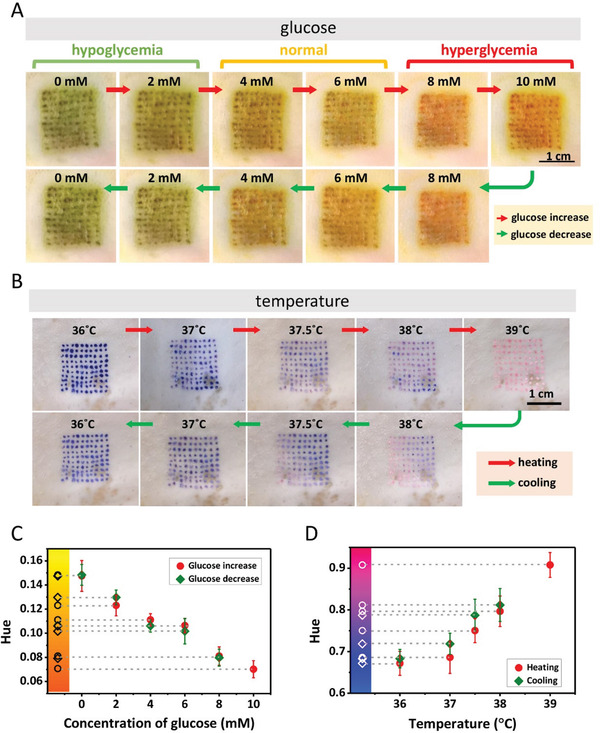
Detections of glucose and body temperature ex vivo using colorimetric dermal tattoo biosensors. A) Detection of glucose ex vivo, in the range of increasing glucose concentration (≈0–10 mm) (upper row) and decreasing glucose concentration (≈10–0 mm) (lower row). B) Detection of temperature ex vivo, during heating (≈36–39 °C) (upper row) and cooling (≈39–36 °C) (lower row). C,D) The Hue values extracted from the images in (A) and (B) as a function of glucose concentration (C) and temperature (D).

To detect glucose ex vivo, the microneedle patches loading with colorimetric glucose sensing reagents were used. We observed that the tattoo color turns out to be green at low glucose levels (hypoglycemia), yellow at normal glucose levels, and red at high glucose levels (hyperglycemia), respectively (**Figure** **4**A). The tattoo glucose sensor shows excellent reversibility of color change when gradually increasing and decreasing the glucose concentration, making it advantageous over conventional strategies based on the colorimetric reaction.^[^ ^9^, ^23^ ^]^ However, the color scale of the detection results varies for abnormal physiological pH values (pH <6.5 or pH >7.5), which could be improved by adding a correction algorithm to adjust color scales according to the pH value for monitoring glucose since the color scales are different at varying pH. Then the tattoo temperature sensor was used to detect temperature ex vivo. It can be seen that the tattoo pattern changes color with changing temperatures (Figure 4B). However, we found that the colors vary at different sites and show nonuniform color distributions, which can be due to the difference in skin thickness and thermal conductivity at different sites of skin. We observed that the tattoo shows negligible color changes when increasing and decreasing temperature to the same initial values, indicating the reversible performance of the fabricated temperature biosensor. Then we used the Hue values to analyze the colorimetric data and found that the Hue values of low, normal, and high glucose levels are in the green, yellow, and red areas, respectively (Figure 4C). What is more, the Hue values decrease with the increase of glucose levels in the range of ≈0–10 mm. As for temperature sensing, the normal and high temperatures as reflected by the Hue values are in the blue and magenta areas, respectively, which increases with increasing temperature (Figure 4D).

### 2.4 Simultaneous Detection of Four Biomarkers In Vivo Using Colorimetric Dermal Tattoo Biosensors

**Figure 5:**
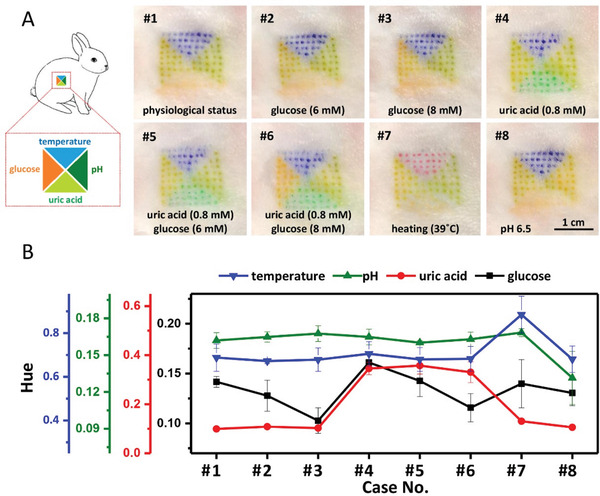
Simultaneous detection of the four biomarkers in vivo using colorimetric dermal tattoo biosensors. A) Schematic illustration (left) and colorimetric detection results (right) for applying microneedle dermal tattoo biosensors for simultaneously detecting multiple biomarkers (i.e., pH, uric acid, glucose, and temperature) in vivo. B) The analysis of images in (A) with Hue. Physiological status: temperature of 36.5 °C, glucose of 4 mm, pH of 7, and uric acid of <0.2 mm. The changed indicators are marked on the images in (A).

Simultaneous detection of multiple health‐related biomarkers by a single tattoo biosensor could greatly improve the efficiency of health management, diagnosis/prognosis accuracy, and reduce operation complexity of users.^[^ ^24^ ^]^ For this, we prepared a microneedle patch with four isolated patterned areas, loaded with colorimetric sensing reagents for four biomarkers (i.e., pH, glucose, uric acid, and temperature). We then used the microneedle patch on the shaved back skin of a rabbit fasting for 12 h. It can be seen that the tattoo clearly formed on the rabbit skin, changing the color under different concentrations of biomarkers injected subcutaneously (**Figure** **5**A). First, the glucose sensing area shows green because of hypoglycemia caused by fasting, while pH, uric acid, and temperature sensing areas show green, orange, and blue, respectively, corresponding to the normal physiological conditions. After subcutaneously injecting glucose solutions under the tattooed skin, we observed obvious color change of the glucose sensing area (i.e., yellow for 6 mm and red for 8 mm), while the colors of other areas remain unchanged. Then, for the uric acid sensing area, it can be seen that the color changes from orange to green with subcutaneously injection of 0.8 mm uric acid. For the temperature sensing area, the color turns from blue to magenta after heating the skin to 39 °C with a hot towel. Last, the color of pH sensing area quickly turns to yellowish brown after injecting PBS solution (pH 6.5) into dermis. The Hue values also confirm the relative changes of biomarkers in skin ISF (Figure 5B). These results indicate that the dermal tattoo biosensor fabricated by the patterned microneedle patch can detect multiple biomarkers in a simultaneous manner.

### 2.5 Monitoring of Biomarkers In Vivo Using Colorimetric Dermal Tattoo Biosensors

**Figure 6:**
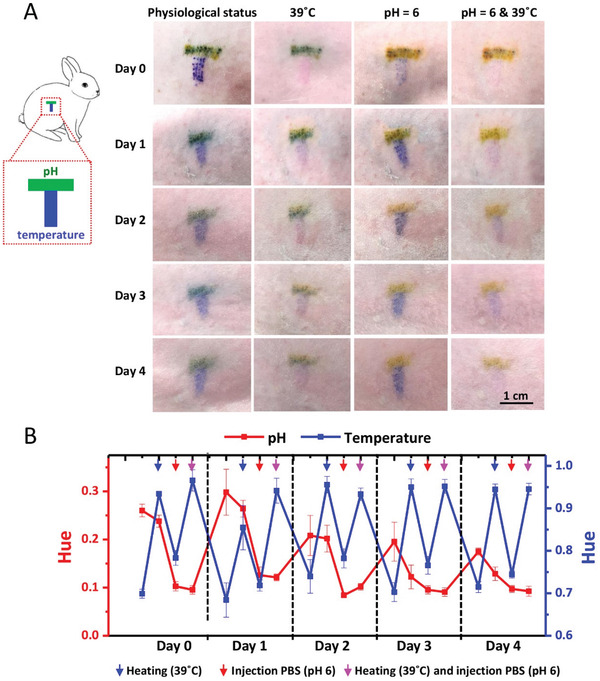
Monitoring of the two typical biomarkers (i.e., pH and temperature) in vivo using colorimetric dermal tattoo biosensors. A) T‐shaped dermal tattoo biosensors (left) and simultaneous monitoring of pH and temperature for 4 days in vivo (right). B) The analysis of images in (A) with the Hue values.

To further demonstrate the application potential of the dermal tattoo biosensor for long‐term health‐care monitoring, we detected the two typical health‐related biomarkers (i.e., pH and temperature) using the dermal tattoo biosensor for several days. We fabricated the dermal tattoo biosensor in shaved rabbit back skin using a “T” patterned microneedle patch loaded with pH indicator and temperature sensing power (**Figure** **6**). We observed that the color of tattoo at pH sensing area changes from green to yellow brown after subcutaneously injecting PBS (pH 6.0) to simulate changes of acid‐base condition in skin ISF. The color of tattoo at the temperature sensing area changes from blue to purple when heating the rabbit skin surface to 39 °C, and immediately reverses to blue once cooling down. Such a phenomenon can be reversibly observed for at least 4 days (Figure 6A). The reversible changes of pH and temperature were further confirmed by the Hue values (Figure 6B). These results indicate that our dermal tattoo biosensor can sense the changes of pH and temperature for at least 4 days. Moreover, we did not observe obvious skin toxicity (e.g., rash, redness, blisters or pimples, raw or cracked areas, and thickening of skin) in the rabbit skin with dermal tattoo biosensor. The H&E slice staining results also show that there is negligible dermal inflammation in tattoo areas, confirming the biocompatibility of the dermal tattoo biosensors (Figure S5, Supporting Information).

In our work, we monitored the changes of biomarkers for a period of time, showing its potential for long‐term health monitoring. Two factors determine the monitoring duration time of the dermal tattoo biosensors for health‐related biomarkers. One is the reversibility of the colorimetric sensing reaction. If the reaction is irreversible, the produced color remains unchanged, even when the biomarker level returns to the original state. In this way, the newly generated signal is influenced and even blocked by the previously produced color. Such an effect is adverse for signal extraction and hinders long‐term monitoring. And when the produced color from high concentration of biomarkers cannot be automatically restored, it is possible to develop external intervention (e.g., local heating or lighting) on skin to reverse the color to the original state since the colorimetric sensing reagents are close to the skin surface (≈1 mm). The other factor is the time scale that tattoo sensing reagents can remain in dermis and show clear color. Since functional failure of sensor can be induced by metabolism of the tattoo sensing reagents, the time scale for the sensing reagents to stay in dermis with clear color deserves to be investigated for prolonging the monitoring duration of tattoo biosensors. Such a time scale is mainly determined by the properties of tattoo sensing reagents, including stability, composition, particle size and shape, state of dispersion, surface area, aggregation state, and surface chemistry.^[^ ^25^ ^]^ Especially, particle size and stability of tattoo ink and its toxicity to important organs have been well studied so far to determine whether the ink can stay in dermis for a long time. The particle size of excellent commercial tattoo ink is between ≈10–5000 nm, making it useful as permanent tattoo inks.^[^ ^26^ ^]^ The particles of ideal tattoo ink could be stable for decades. If pigment particles are degraded into molecules, they lose their color. Thus, colorimetric biosensing reagents encapsulated by polymers and packed as stable micro‐ or nano‐particles have the potential to maintain in dermis for decades and achieve long‐term monitoring of health‐related biomarkers. For some biomarkers that are difficult to achieve continuous long‐term monitoring with colorimetric reactions, microneedle‐fabricated tattoo sensors enable disposable detection, since microneedle patches can easily fabricate new tattoo sensors in a short time, without pains, free of psychological fear, and low cost.

## 3 Conclusions

In this work, we developed a colorimetric dermal tattoo biosensor based on patterned microneedle patches for the detection of multiple health‐related biomarkers without complex and cumbersome operation. The hyaluronic acid hydrogel was used to fabricate microneedle patches for its biocompatibility and rapid degradation in skin for efficient release of sensing reagent. The segmented design of microneedle patch enables it to load multiple sensing reagents to achieve simultaneous detection of multiple biomarkers, including pH, uric acid, glucose, and temperature in vitro and in vivo. The biosensor has been demonstrated with the capability of monitoring multiple biomarkers (i.e., pH and temperature) for at least 4 days, with results read by naked eyes or a camera. The developed colorimetric dermal tattoo biosensor would benefit the field of health management and disease monitoring, especially for chronic patients, because of its aesthetic and minimally invasive properties, suitability for multiple environmental conditions (due to the physical protection of stratum corneum), easy operation, and equipment‐free reading.

## 4 Experimental Section

### Chemicals and Materials

Dimethyl sulfoxide (DMSO), uric acid, and uricase were purchased from Sigma‐Aldrich (USA). Sodium hyaluronate, TMB, thymol blue, methyl red, and phenolphthalein were supplied by Aladdin Co., Ltd. (China). Horseradish peroxidase, glucose oxidase, glucose, and PBS were obtained from MP Biomedicals Co., Ltd. (China). The designed polydimethylsiloxane (PDMS) microneedle patches molds were obtained from Laike Co., Ltd. (China). Temperature responsive power was supplied by Yumingjie Technology Co., Ltd. (China). Polymethyl methacrylate (PMMA) was purchased from Oudifu Co., Ltd. (China). Rabbits were purchased from Xi'an Jiaotong University (China).

### Fabrication of Patterned Microneedle Patches

PMMA plate was cut by laser cutting machine with a designed tattoo pattern and put closely onto the surface of PDMS microneedle negative model to form PDMS/PMMA tattoo microneedle model. Hyaluronic acid (HA) was solved in water to obtain 4% (w/v) solution by constant stirring for 6 h and then added with different color dyes or sensing reagents. The mixture solution was added into PDMS/PMMA tattoo microneedle model, filling up the cavities by centrifugation at 3000 rpm for 20 min. Excess HA outside the micropores was removed. After that, HA microneedle patches were dried at room temperature for 2 days. Then, 4% HA without dyes and reagents was added into the top of micropores and centrifuged at 3000 rpm for 20 min to fill the space at the top of the micropores created by drying of the previously added HA. After drying HA for 1 day at the room temperature, 5% HA without dye was poured onto the surface of dried microneedle patch to fill the PDMS mold and dried at room temperature to form a backboard of microneedle patch. Finally, dried tattoo microneedle patches were meticulously demolded from the PDMS/PMMA mold and stored in a vacuum oven.

### Characterization of Patterned Microneedle Patches

The morphological images of the patterned microneedle patches were captured by a camera and SEM (Hitachi TM 4000 Plus), respectively. The dimension of the microneedles (i.e., height and base size) were measured by ImageJ from SEM images. Patterned microneedle patches were pressed into the shaved rabbit skin by a thumb and kept in the skin for 15 min. After removing the microneedle patches, the images of the tattoo patterns formed in the skin were captured via a camera. The fidelity was calculated by measuring the ratio of the area where the skin dermal tattoo overlapped the designed pattern to the area of entire designed pattern. To measure skin penetration ability and study the forming process of dermal tattoo, the tattooed skin was fixed in 4% paraformaldehyde, sliced and stained by H&E dyes at ≈0–30 days.

### Detection of the Four Biomarkers In Vitro

Tattoo pH sensor was prepared by dissolving 27.8 mg thymol blue, 2.4 mg methyl red, and 17.2 mg phenolphthalein in 500 µL DMSO. 500 µL water was then added into the solution to form the pH indicator of tattoo pH sensor. Tattoo glucose sensor was prepared by adding 20 µL pH indicator, 5 µL glucose oxidase, and 25 µL PBS buffer (10 ×, pH 7.4) in 1 mL water. To detect uric acid, 31 mg TMB was dissolved in 1 mL DMSO to form TMB solution. Enzyme solution was prepared by mixed uricase and horseradish peroxidase (enzyme activity unit ratio = 1:2). Tattoo uric acid sensor was prepared by adding 10 µL TMB solution and 20 µL enzyme solution in 1 mL uric acid solution, and kept at 37 °C for 3 min. Tattoo temperature sensor was prepared by adding 100 mg temperature responsive powder into 1 mL 20% alcohol solution. The photographs of the solution in tubes were captured by a camera and the color changes were quantified by Hue using MATLAB.

### Detection of the Four Biomarkers Ex Vivo

All the animal experiments abided the guidelines of the Institutional Animal Care Committee of Xi'an Jiaotong University. To detect biomarkers ex vivo, colorimetric sensing reagents were mixed with 4% HA to fabricate tattoo microneedle patches. Tattoo microneedle patches were pressed into an isolated and shaved rabbit skin, kept for 15 min and then carefully removed. The skin was soaked into PBS buffer with pH (≈6.0–8.0), uric acid (≈0–1.5 mm), glucose (≈0–10 mm), or water at different temperatures (≈36–39 °C). The tattoo colors in skin were imaged by a camera to analyze the levels of the biomarkers by Hue values.

### Detection of the Four Biomarkers In Vitro

To simultaneously detect pH, uric acid, glucose, and temperature in vivo, rabbits were not fed for 12 h, shaved and epilated on the back skin. Tattoo microneedle patches loaded with colorimetric sensing reagents were pressed into the skin and kept for 15 min. Colorimetric sensing reagents remained in dermal layer and formed tattoo biosensors. To demonstrate the capability of tattoo biosensors for detecting pH, uric acid, and glucose, 1 mL mixture solutions, that is, PBS buffer with pH (≈6.0–8.0), uric acid (≈0–1.5 mm), or glucose (≈0–10 mm), were subcutaneously injected into the tattooed skin. To check the function of tattoo biosensors for measurement of temperature, a hot towel was used to cover the body skin to increase the body temperature and the body surface temperature was recorded by a thermal imager (FOTRIC 288). Photos for tattoo color characterization were recorded by a digital camera immediately after covering the hot towel and the Hue was extracted from photo to analyze the biomarker level. To monitor pH and temperature of the rabbit's skin in vivo, a tattoo microneedle patch loaded with pH and temperature responsible reagents was pressed into rabbit skin for 15 min. After removing the tattoo microneedle patch from the rabbit skin, 200 µL PBS solution with pH 6 was subcutaneously injected into the tattooed skin. Hot towel was used to cover the body skin to increase body surface temperature to 39 °C. Then a camera was used to capture the images of skin tattoo to further analyze the biomarkers levels. The operation was repeated once every day until tattoo disappeared (≈4 days). To study the biocompatibility of dermal tattoo biosensors, tattooed skin was imaged by a camera every day and the skin rash and redness were observed. After 30 days, rabbits were executed and the skin slices were stained with H&E dyes and imaged by an optical microscope to observe inflammation in the tattooed skin dermis.

### Statistical Analysis

The quantitative analysis results of tattoo fidelity in rabbit skin were obtained from three samples. The microneedle dimensions were obtained by analyzing 30 images from three different patches (ten images each patch). Hue values were obtained by analyzing three different microneedle tattoos (100 pinholes per tattoo) in rabbit's skins. The standard deviation of color analysis in the figures are based on patches. Data were expressed as the mean ± standard deviation.

## Conflict of Interest

The authors declare no conflict of interest.

## Supporting information

Supporting Information

## Data Availability

The data that support the findings of this study are available from the corresponding author upon reasonable request.
