# Supplementary material for: A Colorimetric Dermal Tattoo Biosensor Fabricated by Microneedle Patch for Multiplexed Detection of Health‐Related Biomarkers
Source: Adv Sci (Weinh). 2021 Nov 1;8(24):2103030. doi: 10.1002/advs.202103030 (PMC8693053; doi:10.1002/advs.202103030)
Supplement: Supplementary file 1 — Supporting Information [file ADVS-8-2103030-s001.pdf]

© 2021 Wiley-VCH GmbH

ADVANCED SCIENCE
Open Access

Supporting Information

for *Adv. Sci.*, DOI: 10.1002/advs.202103030

A Colorimetric Dermal Tattoo Biosensor Fabricated by Microneedle Patch for Multiplexed Detection of Health-related Biomarkers

Rongyan He[1,2], Hao Liu[1,2], Tianshu Fang[1,2], Yan Niu[1,2], Huiqing Zhang[2,3], Fei Han[1,2], Bin Gao[4], Fei Li[1,2], Feng Xu[1,2]**

Supporting Information

A Colorimetric Dermal Tattoo Biosensor Fabricated by Microneedle Patch for Multiplexed Detection of Health-related Biomarkers

Rongyan He[1,2#], Hao Liu[1,2#], Tianshu Fang[1,2], Yan Niu[1,2], Huiqing Zhang[2,3], Fei Han[1,2], Bin Gao[4], Fei Li[1,2]*, Feng Xu[1,2]*

[1] The Key Laboratory of Biomedical Information Engineering of Ministry of Education, Xi'an Jiaotong University School of Life Science and Technology, Xi'an, China 710049

[2] Bioinspired Engineering and Biomechanics Center (BEBC), Xi'an Jiaotong University, Xi'an, China 710049

[3] Key Laboratory of Thermo-Fluid Science and Engineering of Ministry of Education, School of Energy & Power Engineering, Xi'an Jiaotong University, Xi'an 710049, China

[4] Department of Endocrinology, Tangdu Hospital, Air Force Military Medical University, Xi'an, China 710038

[#] The authors contributed equally

**Corresponding authors: feili@mail.xjtu.edu.cn; fengxu@mail.xjtu.edu.cn*

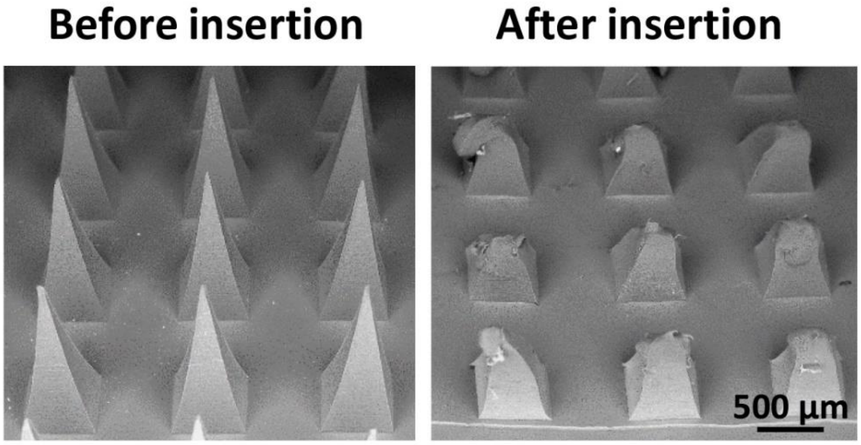

Figure S1. SEM images of the microneedle patch before and after inserting in skin.

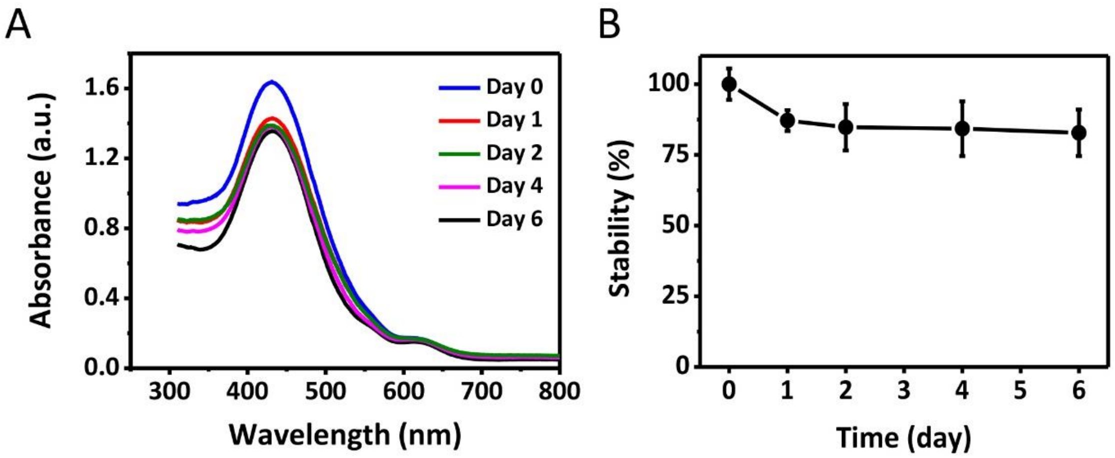

Figure S2. Stability of the pH sensor. (A) Absorbance spectra of pH sensor which reacted with PBS (pH = 7) for different time (0 to 6 days). (B) The stability of the pH sensor after reacting with PBS (pH = 7).

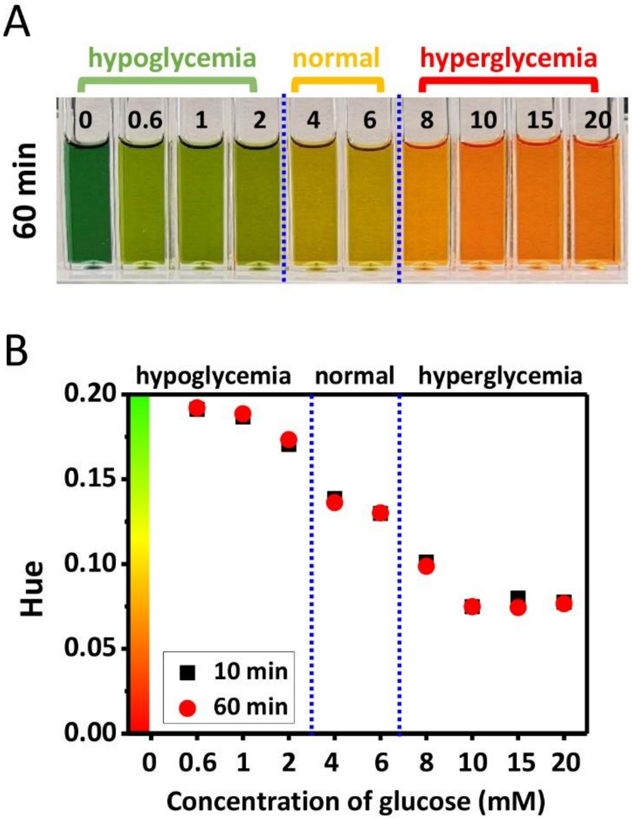

Figure S3. Stability of the glucose colorimetric tattoo biosensor. (A) Images of the glucose indicators after reacting with glucose solution for 60 min. (B) The relationship between the Hue values extracted from the images in (A) and the glucose concentration.

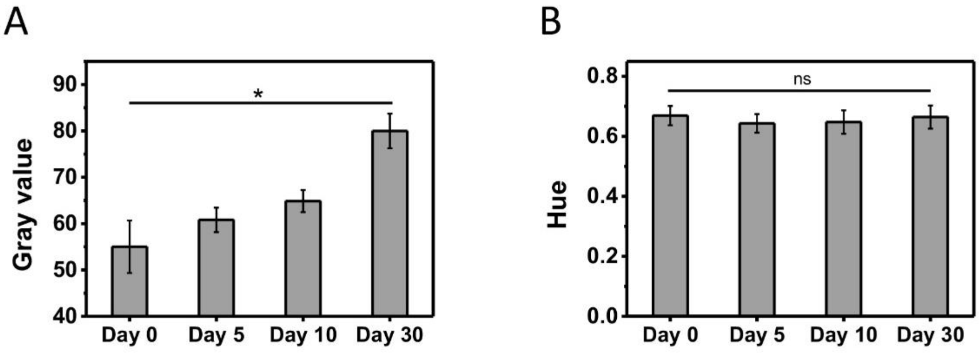

Figure S4. The changes of gray values (A) and Hue (B) of tattoo images in Figure 1D (upper row) with time. "*" means significant difference ($p<0.05$); "ns" means no significant difference ($p>0.05$).

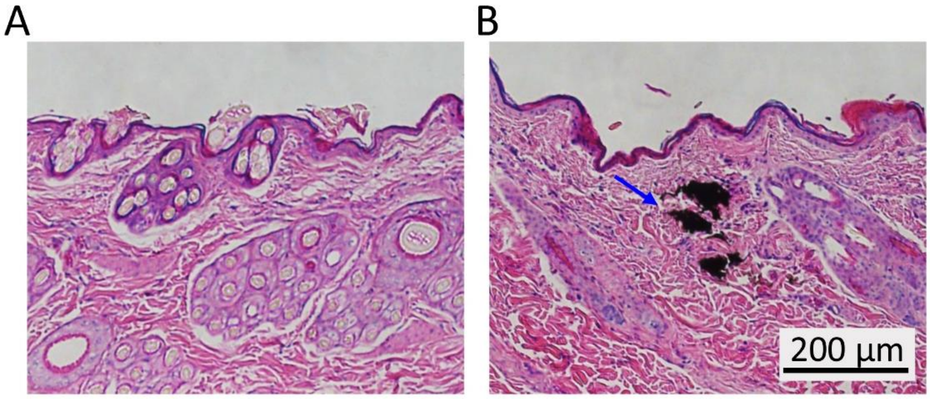

Figure S5. Toxicity of the dermal tattoo biosensor ink. Images of H&E stained skin before (A) and after (B) applying tattoo sensors. The blue arrow points to the biosensor ink.
